# Health and Environmental Co-Benefits of City Urban Form in Latin America: An Ecological Study

**DOI:** 10.3390/su142214715

**Published:** 2022-11-08

**Authors:** Ione Avila-Palencia, Brisa N. Sánchez, Daniel A. Rodríguez, Carolina Perez-Ferrer, J. Jaime Miranda, Nelson Gouveia, Usama Bilal, Andrés F. Useche, Maria A. Wilches-Mogollon, Kari Moore, Olga L. Sarmiento, Ana V. Diez Roux

**Affiliations:** 1 Centre for Public Health, School of Medicine, Dentistry and Biomedical Sciences, Queen’s University Belfast, Belfast BT12 6BA, Northern Ireland, UK; 2 Urban Health Collaborative, Dornsife School of Public Health, Drexel University, Philadelphia, PA 19104, USA; 3 Department of Epidemiology and Biostatistics, Dornsife School of Public Health, Drexel University, Philadelphia, PA 19104, USA; 4 Department of City and Regional Planning, University of California—Berkeley, Berkeley, CA 94720, USA; 5 Institute for Transportation Studies, University of California—Berkeley, Berkeley, CA 94720, USA; 6 CONACYT—National Institute of Public Health, Cuernavaca 62100, Mexico; 7 CRONICAS Center of Excellence in Chronic Diseases, Universidad Peruana Cayetano Heredia, Lima 15074, Peru; 8 School of Medicine, Universidad Peruana Cayetano Heredia, Lima 15102, Peru; 9 Department of Preventive Medicine, University of São Paulo Medical School, São Paulo 01246-903, Brazil; 10 Department of Industrial Engineering, School of Engineering, Universidad de los Andes, Bogotá 111711, Colombia; 11 School of Medicine, Universidad de los Andes, Bogotá 111711, Colombia

**Keywords:** cities, Latin America, population density, air pollution, green space, risk factors, co-benefits

## Abstract

We investigated the association of urban landscape profiles with health and environmental outcomes, and whether those profiles are linked to environmental and health co-benefits. In this ecological study, we used data from 208 cities in 8 Latin American countries of the *SALud URBana en América Latina* (SALURBAL) project. Four urban landscape profiles were defined with metrics for the fragmentation, isolation, and shape of patches (contiguous area of urban development). Four environmental measures (lack of greenness, PM_2.5_, NO_2_, and carbon footprint), two cause-specific mortality rates (non-communicable diseases and unintentional injury mortality), and prevalence of three risk factors (hypertension, diabetes, and obesity) for adults were used as the main outcomes. We used linear regression models to evaluate the association of urban landscape profiles with environmental and health outcomes. In addition, we used finite mixture modeling to create co-benefit classes. Cities with the scattered pixels profile (low fragmentation, high isolation, and compact shaped patches) were most likely to have positive co-benefits. Profiles described as proximate stones (moderate fragmentation, moderate isolation, and irregular shape) and proximate inkblots (moderate-high fragmentation, moderate isolation, and complex shape) were most likely to have negative co-benefits. The contiguous large inkblots profile (low fragmentation, low isolation, and complex shape) was most likely to have mixed benefits.

## 1 Introduction

By 2050, 68% of the world’s population is projected to live in urban areas [1]. The Latin American region is one of the most urbanized regions in the world, with over 500 million people, or 80% of the region’s population, estimated to live in cities [1]. Well-managed urbanization can help maximize the benefits of high levels of population density while minimizing environmental degradation and enhancing sustainability [1].

Urban design and transport planning can positively or negatively influence behaviors and downstream health outcomes. Built environment attributes such as residential density, intersection density, compact development, mixed land use, public transport availability and density, and access to parks can contribute to increasing physical activity levels, mainly through walking [2,3]. Furthermore, walkable environments have been associated with lower body mass index (BMI) and a lower prevalence of diabetes mellitus, and compact communities have been associated with lower cardiovascular morbidity and mortality [4].

Despite health benefits, urban environments can also be a source of environmental hazards for residents [5]. Air pollution concentration tends to be high in cities, especially due to industrial pollution, burning of solid fuels, and gasoline-powered motorized vehicles [6]. Moreover, cities represent intense concentrations of populations and consumption. The IPCC 5th Assessment Report concluded that urban areas generate most carbon emissions from final energy use [7]. Despite consequential research linking urban landscapes to health and environmental outcomes, significant gaps remain. First, most prior research has examined built environment characteristics one at a time, largely ignoring the fact that built environment features are often spatially correlated and that groups of co-occurring features may drive their effects on outcomes. Distinct city characteristics emerge from natural, historical, and policy-related influences [8,9]. Thus, the examination of profiles may be more policy-relevant. Second, most prior research has examined the association between urban landscapes and environmental and health outcomes separately. However, both types of outcomes may cluster because they may be affected by similar built environment features and may be causally related, resulting in health and environmental co-benefits. The concept of co-benefits can be defined as all the positive outcomes (intended and unintended) spanning health and environmental outcomes associated with policies/interventions [10]. Although a planetary health approach has increasingly recognized the interrelatedness of both environmental and health outcomes [11], few, if any, studies have investigated how features of the built environment co-occur and whether these features combined are conducive to better health and environmental co-benefits.

Using rich data on urban form, environmental features, and health outcomes for 208 Latin American cities, we investigated (1) the association of urban landscape profiles with a range of health and environmental outcomes and (2) whether certain urban landscape profiles are linked to environmental and health co-benefits.

## 2 Materials and Methods

### 2.1 Study Design and Population

In this ecological study, we used data from the SALURBAL project, which has compiled and harmonized data on health and social and built environment for cities with a population of more than 100,000 people in 11 Latin American countries. Cities were defined as agglomerations of administrative units (i.e., *municipios, comunas, partidos, delegaciones, cantones*, or *corregimientos*) that are covered, at least in part, by the urban extent of the city; more details are available elsewhere [12].

Of the 371 cities in SALURBAL, we excluded: 1 with no urban landscape city profile data, 5 with missing mortality data, 140 with missing prevalence of risk factors, and 17 with missing social environment data. We included a total of 208 cities in 8 countries (Argentina, Brazil, Colombia, Costa Rica, El Salvador, Guatemala, Mexico, and Peru).

### 2.2 Urban Landscape City Profiles

We use four urban landscape city profiles derived from a prior analysis of city landscape metrics across all SALURBAL countries, described in detail elsewhere [13]. The city profiles were created using finite mixture modeling of six urban landscape metrics, representing subdomains of fragmentation, isolation, and shape of urban development in each city (Supplementary Materials Table S1). Finite mixture modeling uses a mixture of distributions to identify a finite number of classes that maximizes the classification of the units and best represents the heterogeneity of the data [14]. To characterize urban fragmentation, we used: number of urban patches, patch density, area-weighted mean patch size, and effective mesh size. An urban patch was defined as a contiguous area of urban development. Isolation among patches was defined as the mean distance to the nearest urban patch within the city. The area-weighted mean nearest neighbor distance across all patches was used to characterize the city’s isolation. Shape, defined as the compactness and complexity of each patch [15], was assessed using the area-weighted mean shape index. All metrics used the 2012 urban footprint data (in 30 m × 30 m grid cells) from the Global Urban Footprint project [16] and were calculated based on 30 m × 30 m grid cells using the FRAGSTATS 4.2 software package [17].

The four different urban landscape profiles were named according to the characteristics they exhibited: scattered pixels, proximate stones, proximate inkblots, and contiguous large inkblots [13]. The scattered pixels profile includes cities with low fragmentation, high isolation, and compact shape. The proximate stones profile includes cities with moderate fragmentation, moderate isolation, and irregular shape. The proximate inkblots profile includes cities with moderate-high fragmentation (moderate patch density and high patch size), moderate isolation, and complex shape. Finally, the contiguous large inkblots profile includes cities with high fragmentation, low isolation, and complex shape. Supplementary Materials Table S2 contains a graphical description of the profiles, and a visualization tool can be found in the following link: https://salurbal.github.io/profiles/ (accessed on 25 September 2022).

### 2.3 Environmental Outcomes

We examined four environmental outcomes: lack of greenness, PM_2.5_, NO_2_, and carbon footprint. Lack of greenness was created as the complement of exposure to greenness (1-Normalized Difference Vegetation Index (NDVI)). Exposure to greenness was measured as the zonal median of annual maximum NDVI, excluding water. The NDVI data was collected for every year between 2000 and 2016 and averaged. For the analysis, we calculated the mean of the 2002−2016 period. A higher value indicates a higher level of lack of vegetation greenness (or lower level of vegetation greenness).

We obtained air pollution data [PM_2.5_ (μg/m^3^) and NO_2_ (ppb)] from the Atmospheric Composition Analysis Group of Dalhousie University for every year between 1998 and 2016. For the analysis, we calculated the mean of the 2002−2016 period for PM_2.5_ and the mean of the 2002−2012 period for NO_2_. The carbon footprint was measured as carbon emissions per capita in a city. We collected gridded per capita carbon footprint at 1000 m spatial resolution for 2013 from a Gridded Global Model of City Footprints dataset [7]. The per capita carbon footprint unit was tons of CO_2_ emissions/habitant. Higher carbon footprint values indicate greater carbon emissions, which may indicate more emissions of co-pollutants (e.g., NOx, SOx) and anthropogenic heat.

We used the time interval of 2002−2016 for the environmental outcomes to align the data with the dates from which we have data for the different health outcomes explained in the following section.

### 2.4 Health Outcomes

Two types of health outcomes were investigated: mortality rates and risk factors prevalence. We calculated cause-specific mortality rates for non-communicable diseases (NCDs) and non-intentional injuries. We used data from the years 2012−2016 for all countries except for El Salvador, for which we used data from 2010−2014 due to data availability. We obtained mortality data from vital registration systems in each country. Population data were obtained from national census bureaus (or equivalent) that provided population projections or estimations every year from 2010 to 2016 by age and sex for each city unit. We corrected data for the lack of complete registrations of all deaths and redistributed ill-defined causes to specific causes. Causes of death were grouped using the classification for causes of death from the WHO Global Health Estimates (GHE) classification [18]. Non-communicable diseases included all the non-communicable diseases classified in the Global Health Estimates 2015, except malignant neoplasms and other neoplasms. We calculated age-standardized mortality rates for people aged 20 years or older using the 2000−2025 WHO Standard Population as the reference population. More details about how mortality variables were calculated can be found elsewhere [19].

We calculated the prevalence of three chronic disease risk factors among adults aged 20 years or older: hypertension, diabetes, and obesity. For this, we used national surveys conducted in each country. The surveys were conducted in different years in each country: Argentina (2013), Brazil (2013), Colombia (2007), Costa Rica (2005), El Salvador (2014), Guatemala (2002), Mexico (2012), and Peru (2016). Participants were defined as hypertensive if they reported that a physician told them they had hypertension and reported using medications “to lower blood pressure” or to control hypertension as prescribed by a health care provider. A survey participant was classified as having diabetes mellitus if they reported having been told by a physician or health care provider that they had diabetes or high blood sugar levels. A participant was defined as obese if they had a BMI ≥ 30 kg/m^2^, calculated from self-reported (Argentina) or directly measured (all other countries) height and weight. Bayesian hierarchical models stratified by country were used to obtain smoothed prevalence estimates for each survey outcome by sex and age categories for each city. These models use a similar approach as Quick 2020 [20]. We age-standardized the estimates to the 2010 population of all SALURBAL cities, making estimates comparable across cities in different countries (i.e., the prevalence estimates are adjusted for between-city differences in the population distribution).

### 2.5 Covariates

We included a set of additional city-level variables for descriptive or adjustment purposes. City characteristics included sociodemographic features (percentage of females and people older than 65 years), total city population, population density per km^2^, and a city-level social environment index. The social environment index is the mean of the z-scores of four variables: education (% of population who have at least completed primary education among those aged 25 or above), water access (% of households with access to piped water), sanitation (% of households with access to a municipal sewage network), and overcrowding (% of households with more than three people per room reverse coded) [19]. A higher value indicates a better social environment.

### 2.6 Statistical Analyses

We conducted this analysis in three steps. First, we described the distribution of city characteristics, environmental, and health outcomes as well as covariates by urban landscape city profiles, and explored Spearman correlations among environmental and health measures (Supplementary Table S3). For all the models described below, lack of greenness, PM_2.5_, NO_2_, and total population were log-transformed due to their skewed distributions.

Second, we used linear regression models with robust variance to describe the association of urban landscape city profiles with each environmental and health outcome separately. The models were adjusted for the social environment index and country. In secondary analyses, we also adjusted all models for total city population.

Third, we created classes of co-benefits using finite mixture modeling [14]. We included all health and environmental outcomes in the models, and estimated models with 2 to 5 classes in order to choose the number of classes according to interpretability, goodness of fit, and goodness of classification. We defined goodness of fit using the Bayesian Information Criterion (BIC) and Akaike Information Criterion (AIC), with lower values indicating a better fit. We evaluated goodness of classification using entropy, of which a value close to 1 is ideal and above 0.8 is acceptable [21]. We also considered the sample size in each class to ensure that each class included at least 5% of the sample [21]. Based on these criteria, we selected the model with three classes for our co-benefits analysis. All models were adjusted for total population and social environment index. All the outcomes and total population were transformed to z-scores for interpretability in the latent class analysis. Each city was assigned to the class with the highest conditional probability (modal class assignment). We then ran bivariate descriptive analyses to explore the distribution of co-benefit classes by urban landscape profiles.

All models were conducted with a complete case analysis. In all contrasts, a significance value of *p* < 0.05 was considered. All analyses were conducted in Stata version SE 17 (StataCorp LP, College Station, TX, USA).

## 3 Results

Table 1 shows the city characteristics by urban landscape city profiles. The study sample included 208 cities of which 26.4% were in scattered pixels profile, 33.7% in proximate stones profile, 30.8% in proximate inkblots profile, and 9.1% in contiguous large inkblots profile. Cities with a scattered pixels profile were only found in Argentina (25.5%), Colombia (25.5%), Mexico (34.6%), and Peru (14.6%). Half of the proximate stones (47.1%) and proximate inkblots (51.6%) cities were in Mexico, and almost half of the contiguous large inkblots cities (47.4%) were in Brazil. The median city population size was about 400,000 people. In general, city population size increased from scattered pixels to proximate stones, then to proximate inkblots and contiguous large inkblots. The contiguous large inkblots cities were substantially larger than cities in the other profiles, with a median of more than three million inhabitants, and had the highest population density. The city median social environment index increased gradually from scattered pixels (−0.03) to proximate stones, then to proximate inkblots and contiguous large inkblots (0.4), indicating that cities with a contiguous large inkblots profile have a better social environment. Lack of greenness (1-NDVI) was similar across profiles, while air pollution tended to be best in the scattered pixels profile and became progressively worse in proximate stones, proximate inkblots, and contiguous large inkblots (differences across categories only statistically significant for NO_2_). There was a slight gradient in that emissions were lowest in scattered pixels and increased gradually across proximate stones, proximate inkblots, and contiguous large inkblots. There were no clear differences in health outcomes across the profiles, except for higher hypertension prevalence in the contiguous large inkblots and lower NCDs mortality in the scattered pixels and contiguous large inkblots.

Table 2 shows the association of urban landscape city profiles with environmental and health outcomes after adjustment for the social environment index and country. The lack of greenness was lower in cities with proximate stones and contiguous large inkblots profiles than the scattered pixels. Levels of PM_2.5_ and NO_2_ increased progressively from scattered pixels to proximate stones. then to proximate inkblots and contiguous large inkblots. Per capita, carbon emissions were the highest in contiguous large inkblots cities. Non-intentional injury mortality decreased from the scattered pixels to proximate stones city profiles, then to proximate inkblots and contiguous large inkblots. Diabetes prevalence tended to increase, and obesity prevalence tended to decrease from scattered pixels to proximate stones, then to proximate inkblots and contiguous large inkblots.

Table 3 describes the co-benefits classes and shows the distribution of city characteristics, environmental outcomes, and health outcomes across them. We identified three co-benefits classes that described the joint occurrence of environmental and health outcomes. Class 1 (27% of cities) was labeled ‘Positive co-benefits’ because it has favorable values for both environmental and health outcomes, except for PM_2.5_. The percent distribution of cities in Class 1 across countries was 1.80% in Brazil, 55.40% in Colombia, 1.80% in Guatemala, and 41.10% in Peru. Class 2 (29% of cities) was labeled ‘Mixed’ because it has unfavorable outcomes for NO_2_, carbon footprint, and hypertension; intermediate outcomes for diabetes, obesity, and mortality; and favorable outcomes for PM_2.5_. The percent distribution of cities in Class 2 across countries was 52.50% in Argentina, 42.60% in Brazil, 1.60% in Costa Rica, and 3.30% in El Salvador. Class 3 (44% of cities) was labeled ‘Negative co-benefits’ because it presented unfavorable values for all the outcomes, except for PM_2.5_ and hypertension. The percent distribution of cities in Class 3 across countries was 98.90% in Mexico and 1.10% in El Salvador. More details on the three classes can be found in Table S6 and Figure S2 in the Supplementary Materials. In general, cities in the positive co-benefits class tended to be of small or medium size (median population of 347,744 inhabitants) and had higher population density than cities in other classes (13,076 vs. 5755 and 6078 hab/km^2^). Cities in the negative co-benefits class were of medium size (median population of 371,022 habitants), but had lower population density than cities in the positive co-benefits class and had a lower social environment index than cities in other classes (0 vs. 0.3 in both classes 1 and 2). Cities in the mixed class (high emissions and high risk factors class) were larger than other cities (median population of 611,421 inhabitants) and had a population density lower than the cities in the positive co-benefits class.

Table 4 shows the co-benefits classes distribution by urban landscape city profiles. The scattered pixels and proximate stones cities were more likely than the proximate and contiguous large inkblots cities to be in the positive co-benefits class (40% and 31.4% vs. 15.6 and 10.5% respectively). The proximate stones and proximate inkblots cities were more likely than the scattered pixels and contiguous large inkblots cities to be in the negative co-benefits class (48.6% and 51.6% vs. 34.5% and 26.3%). The contiguous large inkblots cities were the least likely to be in the negative co-benefits class (26.3% vs. 34.5%, 48.6%, and 51.6% for scattered pixels, proximate stones, and proximate inkblots, respectively) and the most likely to be in the mixed class (63.2% vs. 25.5%, 20% and 32.8% for scattered pixels, proximate stones, and proximate inkblots respectively).

## 4 Discussion

In this study examining environmental and health outcomes in 208 cities in Latin America, we found that it was possible to identify city profiles that were more likely to show positive or negative health and environmental co-benefits. Overall, 27% of cities fell into the positive co-benefits group, whereas 44% fell into the negative co-benefits group. Cities with positive co-benefits were small to medium-sized cities with higher population density. We also found that cities with one particular urban landscape profile (scattered pixels, representing low fragmentation, high isolation, and more compact development) were more likely than other city profiles to have positive co-benefits. In contrast, the contiguous large inkblot cities (higher fragmentation and complex shape) were the least likely to be in the positive co-benefits class. These cities are generally very large (median population of 3,697,687 inhabitants). The proximate stones (moderate fragmentation of average size and isolated irregular-shaped patches) and proximate inkblots cities (moderate fragmentation of large complex patches) were the cities most likely to be in the negative co-benefits group.

Most of our results align with previous research showing that city characteristics such as low fragmentation, compact shape, and high population density co-occur with improved environmental and health outcomes (co-benefits). Several mechanisms may explain this association. Less fragmented cities may be characterized by shorter distances requiring shorter trips and thus promoting active transport (e.g., walking or cycling). Active transport has been associated with lower air pollution emissions, higher physical activity levels, and lower stress levels [22–24], important determinants of cardiometabolic risk factors [25]. Similarly, walkable environments have been associated with a lower prevalence of diabetes mellitus, and compact communities have been associated with lower cardiovascular morbidity and mortality [4]. Higher population density has also been related to indicators of greater walkability, which could lead to health benefits through higher physical activity levels [3].

Interestingly, the contiguous large inkblot cities, which tended to be the very large and dense cities of the region, were the least likely to be in the positive co-benefits class and tended to predominantly fall into the mixed class (including high emissions and NO_2_, poor or intermediate health outcomes and low PM_2.5_). This result illustrates the need to further explore how these large urban agglomerations, which are growing worldwide, can be better designed and managed to produce positive health and environmental co-benefits.

Our study also highlights the importance of assessing co-benefits. Co-benefits refers to positive outcomes beyond the intended outcome of an intervention or exposure. For example, interventions to change the urban landscape with the goal of reducing emissions can also have health benefits. Analogously, interventions to promote health by increasing active transportation can generate benefits for the environment. The co-benefits concept has been endorsed by the World Health Organization (WHO) since 2011. The 2011 report [26] argues that more compact cities which integrate urban residential and commercial areas enhance environmental and health co-benefits, making walking, cycling and public transport to jobs, schools and services more feasible. The increase of physical activity due to active transport can generate significant health benefits such as preventing certain cancers, type 2 diabetes, heart disease, and other obesity-related risks. In addition, the shift from private motorized transport to walking, cycling and rapid transit/public transport can result in potential health gains such as reduced cardiovascular and respiratory disease from air pollution, less traffic injury, and less noise-related stress. Other interventions such as increasing the area dedicated to greenness in the city can reduce air pollution levels and carbon emissions, with the consequent reduction of mortality rates and risk factors.

Traditional methods in epidemiology tend to identify the independent effects of individual variables, which is a less informative approach in the presence of complexity and clustering of factors. The study of co-benefits highlights the necessity of interdisciplinarity, involving the integration of disciplinary knowledge from different disciplines to develop a better understanding of a problem. It also highlights the need for transdisciplinarity to encourage the co-production of knowledge, incorporating stakeholders, communities, and citizens to provide holistic schemes looking at the dynamics of the urban system. Thus, the relevance of co-benefits supports the value of inter- and transdisciplinary research and also the need for intersectoral collaboration on interventions in both the public health and land-use planning sectors.

Our study has several strengths. First, we explored associations using data from 208 cities spanning 8 countries, providing significant variability in environmental and health outcomes as well as urban landscape features. We used the innovative urban landscape profiles created specifically for Latin American cities. In addition, we included multiple measures of environmental and health-related outcomes, including mortality and risk factors, and used novel approaches to identify clusters of environmental and health outcomes to identify cities with positive and negative co-benefits.

Our study has several limitations. First, the creation of the urban landscape profiles was data-driven, which may limit the generalizability to different contexts. However, our geographic coverage of 208 cities in 8 countries is a considerable strength to represent the urban landscape reality of Latin American cities. Second, the urban landscape metrics were based on cross-sectional data, ignoring potential changes over time. However, these are unlikely to affect our study as urban transformations occur over long periods of time. Despite using highly accurate sources for characterizing the built environment, there may still be measurement errors. Third, while we corrected for potential under-reporting of deaths by estimating completeness at the city level, there is potential for this correction to be insufficient or to be an overcorrection of mortality rates [19]. Moreover, we relied on causes of death as coded on the death certificate, where some are coded as ill-defined. While we redistributed these following previous studies [19], there is the possibility that the proportion of ill-defined death varies by profile. Finally, our risk factor measures were based on self-reported statistics which rely on access to health care, likely resulting in an underestimate of the true prevalence.

## 5 Conclusions

In summary, we identified typologies of cities that performed differently with respect to health and environmental co-benefits. Specifically, we found that cities with more compact shapes, lower fragmentation, high population density, and lower population sizes had more positive health and environmental co-benefits. Our results highlight the need to examine health and environmental outcomes together in a holistic fashion and link these joint outcomes to urban policies. For this, interdisciplinary and transdisciplinary research involving partnerships across health experts, urban planners, and social scientists is critical.

More generally, our results also highlight the need to recognize and highlight the interconnections and interrelatedness of the Sustainable Development Goals (SDGs) identified as part of the United Nations 2030 Agenda for Sustainable Development. Specifically, our results show the link between SDG3 (ensure healthy lives and promote well-being all at all ages) and SDG 11 (make cities and human settlements inclusive, safe, resilient, and sustainable). Explicit recognition and analyses of these interrelations (including an understanding and documentation of co-benefits) is needed to identify the best policy options to make cities both healthy and environmentally sustainable.

## Supplementary Material

Supplementary Material

## Figures and Tables

**Table 1:** Characteristics of the studied cities by urban landscape profiles.

	Total	Scattered Pixels	Proximate Stones	Proximate Inkblots	Contiguous Large Inkblots	*p*-Value *
	p50 (iqr)	p50 (iqr)	p50 (iqr)	p50 (iqr)	p50 (iqr)	
Number of cities	208	55	70	64	19	
City characteristics						
Sociodemographic characteristics						
Total population (hab)	407,845.4 (684,555.4)	196,360 (117,804)	323,763.6 (212,514)	899,117(511,916)	3,697,687 (6,483,998)	<0.001
Population density (hab/km^2^)	7010.4 (4567.9)	7063 (7801)	7062 (4682.1)	6615 (3338)	7442.4 (4258.6)	0.655
Census age ≥ 65 years (%)	10.2 (3.1)	10.5 (3.5)	10.2 (3.8)	9.7 (2.3)	10.7 (2.3)	0.127
Census females (%)	52.5 (1.7)	52.1 (1.9)	52.4 (1.9)	52.7 (1.5)	52.9 (0.9)	0.021
Social Environment Index	0.1 (0.9)	−0.03 (1.3)	0.1 (1.0)	0.1 (0.7)	0.4 (0.4)	0.015
Urban Landscape Metrics						
Total built-up area (hectares)	5967.7(11,069.4)	2798.3 (2486.4)	4214.9 (3353)	14,240 (9041)	55,997.5 (27,081.8)	<0.001
Number of Urban Patches (N)	485 (726.5)	276 (349)	363 (242)	954 (634.5)	2888 (2281)	<0.001
Patch Density (N/km^2^)	0.3 (0.5)	0.1 (0.1)	0.3 (0.3)	0.4 (0.4)	0.8 (0.3)	<0.001
Area-weighted Mean Patch Size (km^2^/N)	2372 (4452)	1082.2 (630.6)	1950.6 (1705.3)	6052.2 (5526.8)	24,567.9 (19,677.7)	<0.001
Effective Mesh Size (km^2^)	78.2 (286.6)	8.6 (11)	57.5 (66.5)	313.5 (560)	3319.1 (6472.9)	<0.001
Area-weighted Mean Shape Index (from 1 to infinity)	5.2 (2.4)	4.2 (1)	5 (1.4)	6.6 (2.2)	9.6 (3)	<0.001
Area-weighted Mean Nearest Neighbor Distance (m)	83.1 (39.1)	122 (65.8)	80.3 (19)	76.6 (27.4)	65.4 (5.3)	<0.001
Other measures						
Country						<0.001
Argentina	15.4%	25.5%	12.9%	10.9%	10.5%	
Brazil	13.0%	0.0%	5.7%	21.9%	47.4%	
Colombia	14.9%	25.5%	17.1%	6.3%	5.3%	
Costa Rica	0.5%	0.0%	0.0%	0.0%	5.3%	
El Salvador	1.4%	0.0%	2.9%	1.6%	0.0%	
Guatemala	0.5%	0.0%	0.0%	1.6%	0.0%	
Mexico	43.3%	34.6%	47.1%	51.6%	26.3%	
Peru	11.1%	14.6%	14.3%	6.3%	5.3%	
Environmental outcomes						
Lack of greenness (1-NDVI)	0.2 (0.2)	0.2 (0.3)	0.2 (0.2)	0.2 (0.2)	0.2 (0.1)	0.503
PM_2.5_ (μg/m^3^)	16.7 (7.8)	15.8 (6.0)	17.6 (5.4)	16.2 (9.5)	17.4 (8.7)	0.294
NO_2_ (ppb)	0.2 (0.3)	0.2 (0.2)	0.3 (0.2)	0.3 (0.4)	0.6 (1.5)	<0.001
Carbon footprint (CO_2_ emissions/hab)	3.8 (1.5)	3.8 (1.8)	3.8 (1.6)	3.9 (1.3)	3.8 (1.4)	0.786
Health outcomes (age adjusted)						
NCDs mortality rate (per 100,000 hab)	524.9 (155.8)	489.2 (160.1)	536.2 (150)	557.2 (135.1)	495.7 (142.8)	0.045
Non-intentional injuries mortality rate (per 100,000 hab)	42.3 (13.4)	42.2 (14)	42.7 (12.1)	44.1 (14.3)	38.3 (14.8)	0.210
Hypertension prevalence (%)	10.8 (3.7)	10.1 (4.8)	10.4 (3.2)	11.5 (3.4)	13.8 (7.1)	<0.001
Diabetes prevalence (%)	7.7 (4)	6.4 (5)	8 (4.3)	7.8 (3.1)	6.5 (3.1)	0.330
Obesity prevalence (%)	26.5 (12.9)	23 (14.9)	29.1 (13.9)	28.9 (12.6)	23 (10.2)	0.216

* iqr, interquartile range; NDVI, Normalized Difference Vegetation Index; PM_2.5_, Particulate Matter that have a diameter of less than 2.5 μm; NO_2_, Nitrogen dioxide; CO_2_, Carbon dioxide; SD, standard deviation. * Chi^2^ test for categorical variables, Kruskal Wallis test for continuous variables, comparing whether proportions or medians are similar across groups.

**Table 2:** Associations of environmental and health outcomes with urban landscape profiles adjusted for social environment and country.

Urban Landscape Profiles	Scattered Pixels	Proximate Stones	Proximate Inkblots	Contiguous Large Inkblots
Environmental outcomes		Coef (95% CI)	Coef (95% CI)	Coef (95% CI)
Lack of greenness (1-NDVI) ^a^	0 (Reference)	−24.16 (−49.27, −3.27) *	−15.39 (−43.23, 7.57)	−26.55 (−59.48, −0.00) *
PM_2.5_ (μg/m^3^)^a^	0 (Reference)	17.46 (5.35, 30.97) *	21.58 (6.72, 38.50)*	40.91 (17.45, 69.05)*
NO_2_ (ppb) ^a^	0 (Reference)	23.54 (−3.07, 57.32)	34.53 (2.83, 75.99) *	363.94 (187.01, 649.94) *
Carbon footprint (CO_2_ emissions/hab)	0 (Reference)	−0.08 (−0.18, 0.03)	−0.12 (−0.25, 0.00)	0.13 (−0.12, 0.39)
Health outcomes (age adjusted)				
NCDs mortality rate (per 100,000 hab)	0 (Reference)	9.08 (−18.6, 36.76)	3.53 (−23.29, 30.34)	−4.30 (−39.68, 31.08)
Non-intentional injuries mortality rate (per 100,000 hab)	0 (Reference)	−3.95 (−9.21,1.3)	−4.43 (−9.92,1.07)	−12.15 (−18.41, −5.9)*
Hypertension prevalence (%)	0 (Reference)	−0.04 (−0.52, 0.44)	0.26 (−0.31, 0.84)	0.21 (−0.68,1.09)
Diabetes prevalence (%)	0 (Reference)	0.20 (−0.12,0.52)	0.22 (−0.14, 0.57)	0.58 (0.11, 1.05) *
Obesity prevalence (%)	0 (Reference)	−0.99 (−2.61, 0.64)	−1.38 (−3.2, 0.43)	−2.03 (−3.99, −0.07) *

* *p* < 0.05.

a Log-transformed variables. The results are reported as % of increase of the outcomes comparing each profile to the reference (scattered pixels). To do this, we applied the following formula: (exp(coef)-1) × 100. NDVI, Normalized Difference Vegetation Index; PM_2.5_, Particulate Matter that have a diameter of less than 2.5 μm; NO_2_, Nitrogen dioxide; CO_2_, Carbon dioxide; NCDs, non-communicable diseases. Linear regression models adjusted for social environment index and country.

**Table 3:** Description of co-benefits classes and city characteristics by class.

Class Number	Class 1	Class 2	Class 3	*p*-Value *
Co-benefits class name	Positive co-benefits	Mixed	Negative co-benefits	
Description of co-benefits class	Positive health & environmental co-benefits, except PM_2.5_	High NO_2_ & carbon footprint, high hypertension intermediate mortality, diabetes and obesity, low PM_2.5_	Negative health & environmental co-benefits, except PM_2.5_ and hypertension	
City characteristics	p50 (iqr)	p50 (iqr)	p50 (iqr)	
Number of cities	56	61	91	
Total population (hab)	347,743.5 (451,903)	611,421 (1,373,158)	371,021.6 (610,151.4)	0.019
Population density (hab/km^2^)	13,075.6 (6380.5)	5755.3 (2966.6)	6077.6 (2003.1)	<0.001
≥65 years (%)	10.2 (3.1)	12 (3.8)	9.5 (1.9)	<0.001
Females (%)	52.6 (2.3)	52.8 (1.4)	52.4 (1.5)	0.117
Social Environment Index	0.3 (0.8)	0.3 (0.6)	0 (1)	0.045
Country Argentina	0%	52.50%	0%	<0.001
Brazil	1.80%	42.60%	0%	
Colombia	55.40%	0%	0%	
Costa Rica	0%	1.60%	0%	
El Salvador	0%	3.30%	1.10%	
Guatemala	1.80%	0%	0%	
Mexico	0%	0%	98.90%	
Peru	41.10%	0%	0%	
Environmental outcomes				
Lack of greenness (1-NDVI)	0.2 (0.3)	0.2 (0.1)	0.2 (0.2)	0.468
PM_2.5_ (μg/m^3^)	18.9 (7.9)	14.2 (6.4)	16.3 (8.1)	<0.001
NO_2_ (ppb)	0.2 (0.1)	0.3 (0.3)	0.3 (0.4)	<0.001
Carbon footprint (CO_2_ emissions/hab)	2.6 (0.5)	4.5 (1.8)	4.2 (0.5)	<0.001
Health outcomes (age adjusted)				
NCDs mortality	401 (127.5)	489.2 (73.2)	606.3 (76.2)	<0.001
Non-intentional injuries mortality	38.5 (18.8)	42.2 (14.8)	45.5 (11.5)	<0.001
Hypertension	7.4 (1.8)	14.1 (2.1)	10.7(1.6)	<0.001
Diabetes	3.8 (1.1)	6.5 (2)	8.8 (1.1)	<0.001
Obesity	18.1 (7.9)	21.3 (3.2)	33.6 (6.3)	<0.001

* Chi^2^ test for categorical variables, Kruskal Wallis test for continuous variables, comparing whether proportions or medians are similar across groups.

**Table 4:** Co-benefits classes distribution by city profiles.

	Scattered Pixels	Proximate Stones	Proximate Inkblots	Contiguous Large Inkblots	Total	*p*-Value *
Co-benefits class						0.001
Class 1: Positive co-benefits	40.0%	31.4%	15.6%	10.5%	26.9%	
Class 2: Mixed	25.5%	20.0%	32.8%	63.2%	29.3%	
Class 3: Negative co-benefits	34.5%	48.6%	51.6%	26.3%	43.8%	
Total	100%	100%	100%	100%	100%	

* Chi^2^ test comparing whether proportions are similar across groups. Percentages are column percentages.

## Data Availability

The SALURBAL project welcomes queries from anyone interested in learning more about its dataset and potential access to data. To learn more about SALURBAL’s dataset, visit the SALURBAL project website or contact the project at salurbal@drexel.edu. After publication of this study, study protocols, data dictionaries, and requested study data may be made available to interested investigators after they have signed a data use agreement with SALURBAL and if their study proposal, developed in collaboration with SALURBAL investigators, is approved by the SALURBAL proposal and publications committee. Some data may not be available to external investigators because of data confidentiality agreements.
