# Supplementary material for: Health and Environmental Co-Benefits of City Urban Form in Latin America: An Ecological Study
Source: Sustainability. Author manuscript; Available in PMC 2023 Mar 15. (PMC7614319; doi:10.3390/su142214715)
Supplement: Supplementary Material [file EMS156784-supplement-Supplementary_Material.pdf]

sustainability

MDPI

Table S1. Urban landscape subdomains and metrics used to create urban landscape profiles.

Subdomains	Metric	Abbreviation	Formula	Description	Icon
Area	Total urban area	TUA	$TUA_i = \sum_{j \in i, j \in urban} A_j$	*Aj* refers to the area of 30m × 30m gridcell *j* within the geographic unit *i* and categorized as urban.	NA
Fragmentation	Number of Urban Patches (N)	NUP	NA	NA	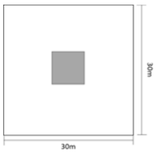 low 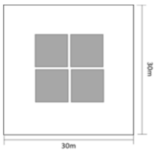 high
	Patch Density (N/km^2)	PD	$PD_i = \frac{NUP_i}{TUA_i}$	NA	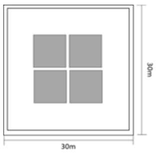 low 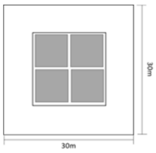 high
	Area-weighted Mean Patch Size (km^2/N)	AWMPS	$AWMPS_i = \frac{\sum_{j \in i} \frac{UA_j^2}{TUA_i}}{NUP_i}$	Where UA_j refers to the area of urban patch *j* located in the city *i*.	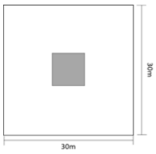 low 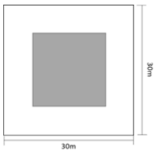 high
	Effective Mesh Size (km^2)	EMS	$EMS_i = \frac{\sum_{k \in i} UA_k^2}{TUA_i}$	Where UA_j refers to the area of urban patch *j* located in the city *i*.	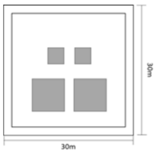 low 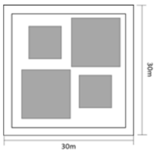 high
Isolation	Area-weighted Mean Nearest Neighbor Distance (meters)	AWMNND	$AWMNND_i = \frac{\sum_{k \in i} \frac{NNHG_k * UA_k}{TUA_i}}{NUP_i}$	Where $NNGH_k$ is the nearest neighbor distance of urban patch *k* in city *i*, UA_k refers to the area of urban patch *k* located in the city *i*	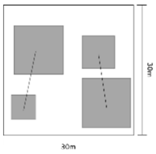 low 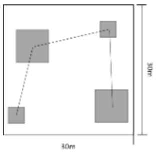 high
Shape	Area-weighted Mean Shape Index	AWMSI	$AWMSI_i = \frac{\sum_{k \in i} \frac{SHPINDX_k * UA_k}{TUA_i}}{NUP_i}$	Where $SHPINDX_k$ refers to the shape index of urban patch *k* inside city *i*, specifically, shape index is the ratio of the actual perimeter of a patch to the minimum perimeter possible for a maximally compact patch with the same size. UA_k refers to the area of urban patch *k* located in the city *i*.	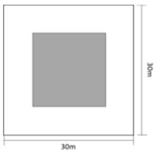 low 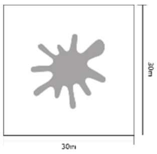 high

Table S2. Urban Landscape profiles.

Label	Description	Caption	City example
Scattered pixels	Cities with low patch density and low area weighted mean patch size, high isolation, and patches with compact shape.	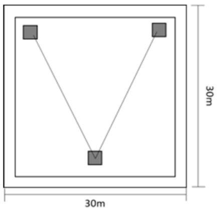	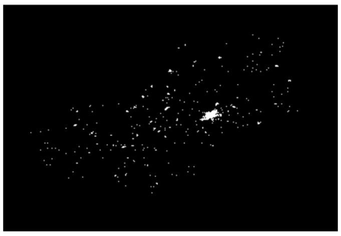 Fresnillo, Mexico (204192)
Proximate stones	Cities with moderate patch density and moderate area weighted mean patch size, moderate isolation, and patches with irregular shape.	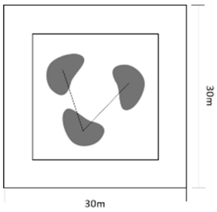	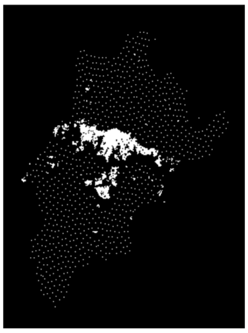 Pocos de Caldas, Brazil (102146)
Proximate ink-blots	Cities with moderate patch density and high area weighted mean patch size, moderate isolation, and patches with complex shape.	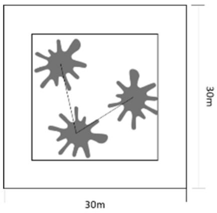	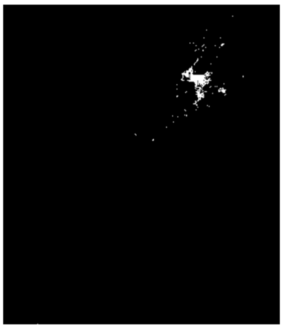 Cartagena, Colombia (104104)
Contiguous large inkblots	Cities with high patch density and high area weighted mean patch size, low isolation, and patches with complex shape.	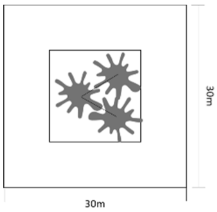	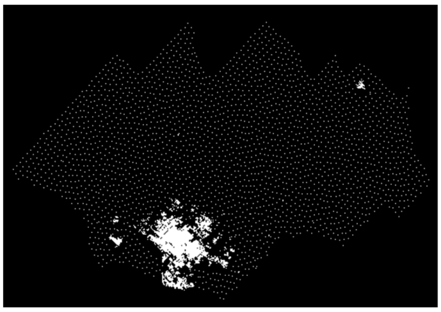 Buenos Aires, Argentina (101101)

Table S3. Correlations between outcomes (Spearman).

	Lack of greenness	$PM_{2.5}$	NO_2	Carbon footprint	NCDs mortality	Non-intentional injuries mortality	Hypertension	Diabetes	Obesity	Total population	Social Environment Index
Environmental outcomes											
Lack of greenness (1-NDVI)	1										
$PM_{2.5}$ (μg/m^3)	−0.1334	1									
NO_2 (ppb)	0.2702	0.0031	1								
Carbon footprint (CO_2 emissions/hab)	0.2671	−0.3366	0.4877	1							
Health outcomes											
NCDs mortality*	0.0293	−0.1988	0.2484	0.3696	1						
Non-intentional injuries mortality*	0.1619	−0.248	−0.0889	−0.0093	0.3336	1					
Hypertension**	0.0898	−0.343	0.2961	0.4689	0.2081	0.0679	1				
Diabetes**	0.1001	−0.1761	0.3827	0.6065	0.6665	0.1074	0.387	1			
Obesity**	0.2171	−0.1622	0.2158	0.3496	0.6717	0.3063	0.0942	0.6818	1		
Total population	−0.0533	−0.0449	0.2012	−0.0424	−0.0127	−0.1445	0.2494	−0.0064	−0.0726	1	
Social Environment Index	0.3968	−0.2365	0.3542	0.3221	−0.2492	−0.1217	0.0691	−0.1026	−0.1389	0.181	1

*Mortality rates per 100,000 hab adjusted by age.

**Prevalences, median

Table S4. Associations of environmental and health outcomes with urban landscape profiles adjusted for social environment, country and total population.

Urban landscape profiles	Scattered pixels	Proximate stones	Proximate inkblots	Contiguous large ink-blots
Environmental outcomes		Coef (95% CI)	Coef (95% CI)	Coef (95% CI)
Lack of greenness (1-NDVI)[a]	0 (Ref.)	−22.68 (−49.81, −0.46)*	−10.43 (−47.88, 21.27)	−15.34 (−81.82, 36.68)
$PM_{2.5}$ (μg/m^3) [a]	0 (Ref.)	16.71 (4.08, 30.87)*	18.74 (−2.82, 44.98)	34.07 (−3.20, 85.50)
NO_2 (ppb) [a]	0 (Ref.)	15.69 (−13.49, 51.89)	5.67 (−45.03, 61.94)	178.76 (29.23, 501.32)*
Carbon footprint (CO_2 emissions/hab)	0 (Ref.)	−0.07 (−0.18, 0.04)	−0.12 (−0.29, 0.06)	0.15 (−0.22, 0.51)
Health outcomes				
NCDs mortality	0 (Ref.)	14.68 (−11.99, 41.35)	24.1 (−8.57, 56.77)	39.1 (−20.23, 98.43)
Non-intentional injuries mortality	0 (Ref.)	−0.89 (−6.14, 4.36)	6.83 (−0.4, 14.05)	11.59 (0.27, 22.91)*
Hypertension	0 (Ref.)	0.01 (−0.49, 0.51)	0.43 (−0.43, 1.29)	0.56 (−1.13, 2.25)
Diabetes	0 (Ref.)	0.2 (−0.14, 0.54)	0.23 (−0.29, 0.74)	0.6 (−0.23, 1.42)
Obesity	0 (Ref.)	−1 (−2.68, 0.68)	−1.42 (−3.78, 0.93)	−2.12 (−5.75, 1.51)

*$p<0.05$; [a] Log-transformed variables. The results are reported as % of increase of the outcomes for every one-unit increase in the exposure. To do that, we applied the following formula: (exp(coef)-1) × 100.; $PM_{2.5}$, Particulate Matter that have a diameter of less than 2.5 micrometers. NO_2, Nitrogen dioxide. CO_2, Carbon dioxide; Linear regression models adjusted by climate zones, social environment index, country.

Table S5. Description of the latent class analysis for 2 classes.

	Class 1	Class 2
Probability of Class	0.36	0.64
Environmental outcomes		
Lack of green space	−0.17	0.09
$PM_{2.5}$	0.39	−0.22
NO_2	−0.57	0.31
Carbon footprint per capita	−1.08	0.60
Health outcomes		
NCDs mortality	−0.82	0.45
Non-intentional injuries mortality	−0.04	0.02
Hypertension	−0.63	0.35
Diabetes	−1.16	0.64
Obesity	−0.77	0.43

All classes were adjusted by social environment index, total population.

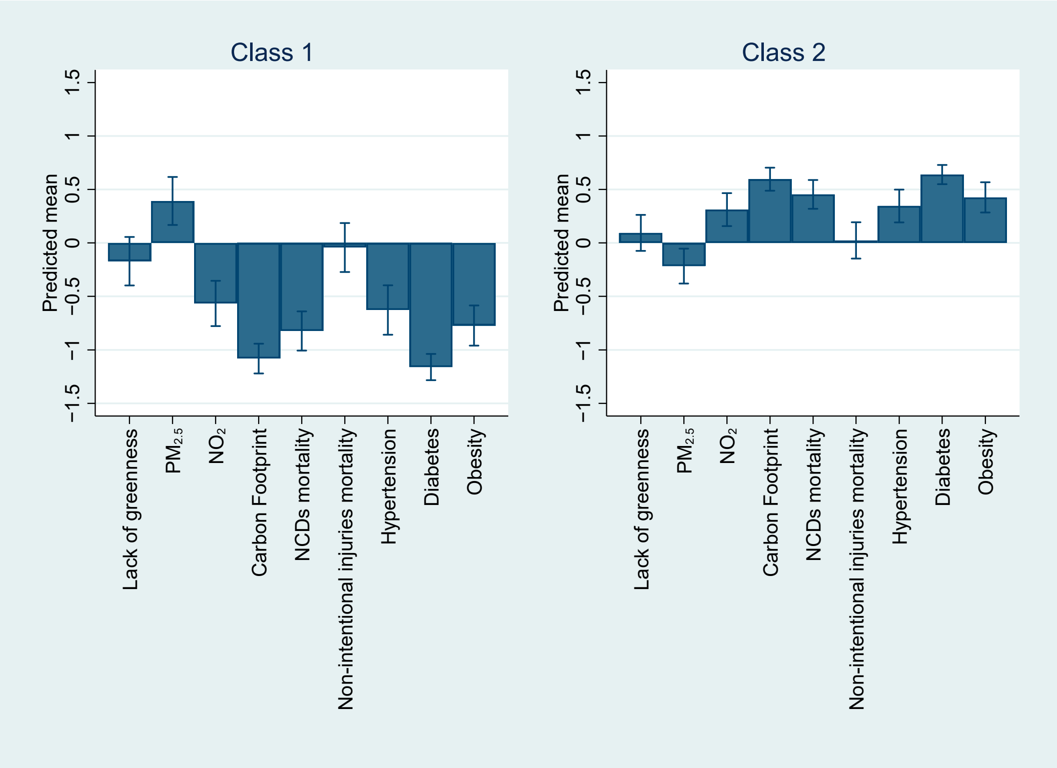

Figure S1. Description of the latent class analysis for 2 classes. All classes were adjusted by social environment index, total population.

Table S6. Description of the latent class analysis for 3 classes.

	Class 1	Class 2	Class 3
Probability of Class	0.27	0.29	0.44
Environmental outcomes			
Lack of green space	−0.05	−0.08	0.09
$PM_{2.5}$	0.65	−0.25	−0.24
NO_2	−0.54	0.07	0.29
Carbon footprint per capita	−1.11	0.43	0.41
Health outcomes			
NCDs mortality	−1.04	−0.22	0.80
Non-intentional injuries mortality	−0.14	−0.13	0.17
Hypertension	−1.17	1.15	−0.04
Diabetes	−1.36	0.02	0.83
Obesity	−0.83	−0.61	0.92

All classes were adjusted by social environment index, total population.

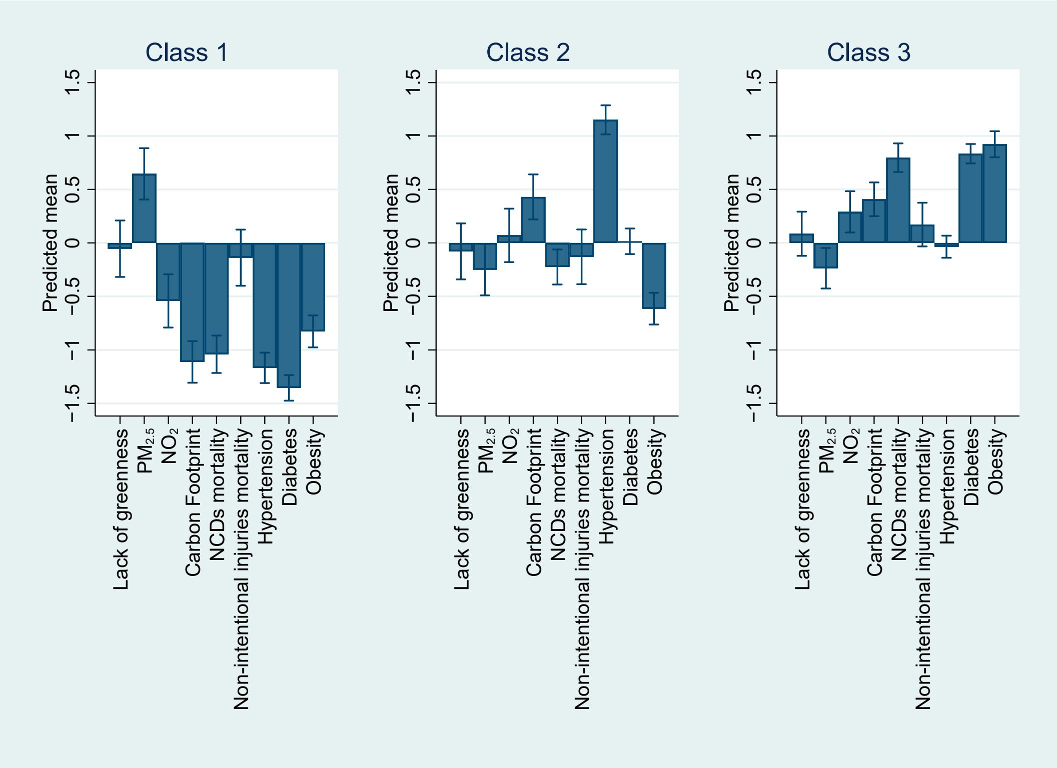

Figure S2. Description of the latent class analysis for 3 classes. All classes were adjusted by social environment index, total population.

Table S7. Description of the latent class analysis for 4 classes.

	Class 1	Class 2	Class 3	Class 4
Probability of Class	0.26	0.15	0.16	0.43
Environmental outcomes				
Lack of green space	−0.01	−0.55	0.30	0.09
$PM_{2.5}$	0.62	−0.23	−0.14	−0.24
NO_2	−0.54	−0.36	0.42	0.29
Carbon footprint per capita	−1.10	−0.79	1.44	0.41
Health outcomes				
NCDs mortality	−1.11	−0.11	−0.30	0.81
Non-intentional injuries mortality	−0.20	0.29	−0.45	0.18
Hypertension	−1.22	1.22	0.99	−0.05
Diabetes	−1.39	−0.30	0.30	0.83
Obesity	−0.83	−0.54	−0.67	0.94

All classes were adjusted by social environment index, total population.

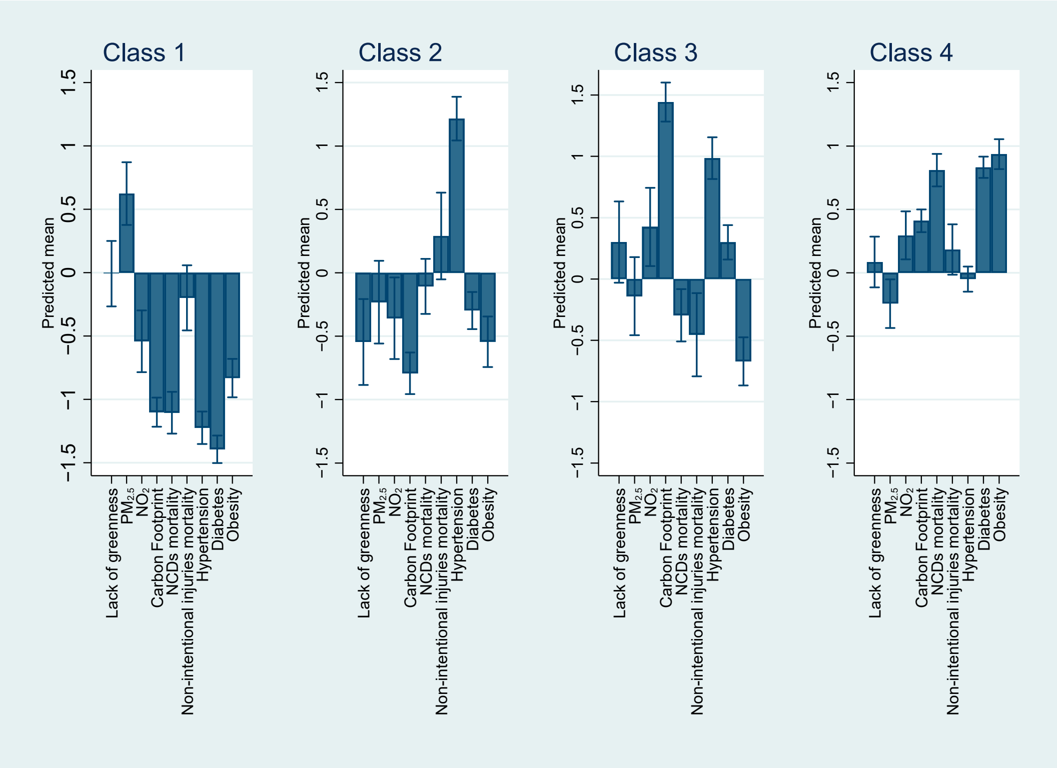

Figure S3. Description of the latent class analysis for 4 classes. All classes were adjusted by social environment index, total population.

Table S8. Description of the latent class analysis for 5 classes.

	Class 1	Class 2	Class 3	Class 4	Class 5
Probability of Class	0.26	0.15	0.31	0.16	0.12
Environmental outcomes					
Lack of green space	−0.01	−0.54	−0.36	0.30	1.18
$PM_{2.5}$	0.62	−0.24	0.24	−0.13	−1.46
NO_2	−0.54	−0.36	0.29	0.43	0.29
Carbon footprint per capita	−1.10	−0.79	0.29	1.44	0.69
Health outcomes					
NCDs mortality	−1.10	−0.10	0.72	−0.29	1.03
Non-intentional injuries mortality	−0.20	0.30	0.01	−0.45	0.60
Hypertension	−1.22	1.22	−0.12	0.98	0.13
Diabetes	−1.39	−0.31	0.85	0.30	0.81
Obesity	−0.83	−0.55	0.86	−0.66	1.14

All classes were adjusted by social environment index, total population.

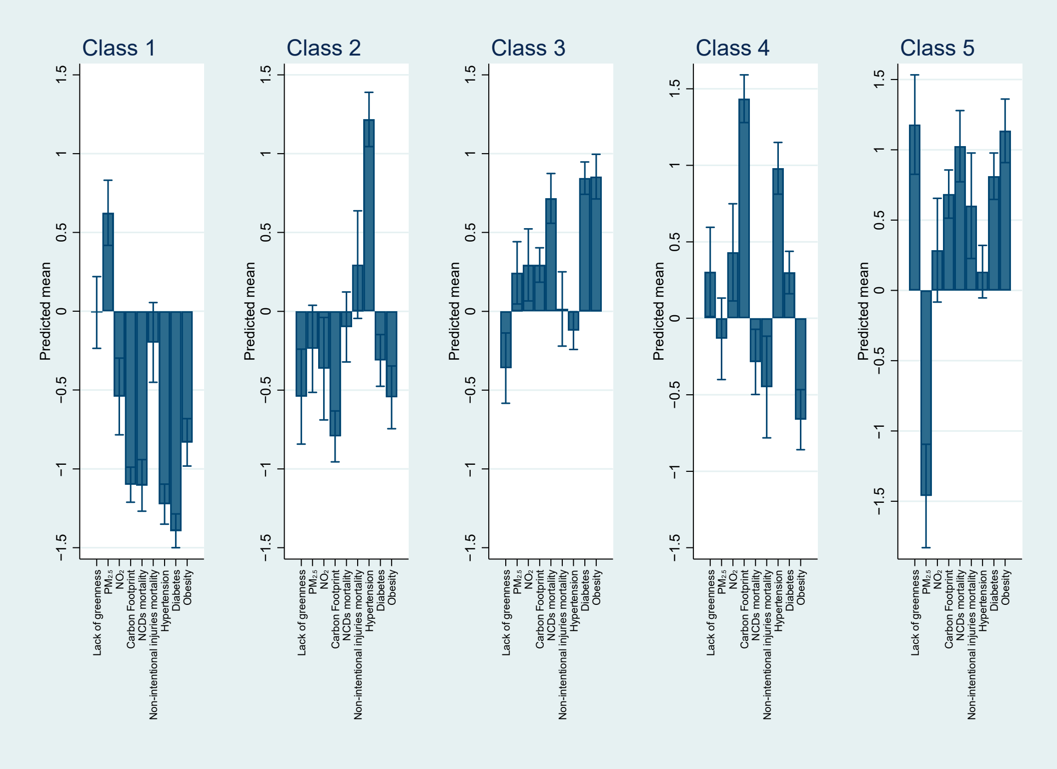

Figure S4. Description of the latent class analysis for 5 classes. All classes were adjusted by social environment index, total population.

Table S9. Latent Class Analysis Model fit criteria.

Models	N	LL (null)	LL (model)	df	AIC	BIC
2 Class	208	.	−2383.013	30	4826.027	4926.153
3 Class	208	.	−2231.721	42	4547.442	4687.618
4 Class	208	.	−2098.637	54	4305.273	4485.5
5 Class	208	.	−2048.533	66	4229.066	4449.343

Table S10. Latent Class Analysis Model Diagnostic criteria.

Models	Smallest class count (n)	Smallest class size (%)	Entropy
2 Class	74	35.58	0.963
3 Class	56	26.92	0.965
4 Class	32	15.38	0.994
5 Class	25	12.02	0.992
